# Salivary metabolic profile in patients with oral squamous cell carcinoma and potentially malignant disorders

**DOI:** 10.1007/s00784-026-07192-0

**Published:** 2026-10-02

**Authors:** Lauriina Soiniemi, Eelis Hyvärinen, Jopi Mikkonen, Heli Jäsberg, Bina Kashyap, Arja Kullaa

**Affiliations:** 1 Institute of Dentistry, School of Medicine, University of Eastern Finland, Kuopio campus, Yliopistonranta 8, Kuopio, FI- 70210 Finland; 2 Educational Dental Clinic, Kuopio University Hospital, The Wellbeing Services County of North Savo, Kuopio, 70210 Finland; 3 Department of Technical Physics, University of Eastern Finland, Kuopio, Finland; 4 Oral pathology department, Agartala Government Dental College, Agartala, West Tripura 799001 India

**Keywords:** Oral potentially malignant lesions, Oral squamous carcinoma, Saliva, Metabolites, NMR spectroscopy

## Abstract

**Objectives:**

Oral Potential Malignant Lesions (OPMDs), including oral leukoplakia (OLK) and lichen planus (OLP), as well as Oral Squamous Cell Carcinoma (OSCC) are influenced by oral dysbiosis and might represent a disease continuum. Our hypothesis was that the oral salivary metabolomic reflects the association of those diseases. Hence, we aimed to study and compare salivary metabolites and metabolic pathways in OPMDs and OSCC.

**Material and methods:**

Unstimulated saliva samples were collected from 60 participants: OLK (*n* = 15), OLP (*n* = 15), OSCC (*n* = 15), and healthy controls (HCs; *n* = 15). Metabolites were quantified using NMR spectroscopy, and pathway analyses were performed with MetaboAnalyst 6.0.

**Results:**

Four metabolites (acetate, taurine, pyruvate, methylamine) were elevated, while proline was consistently decreased in all disease groups relative to HCs. Four key pathways were altered: pyruvate metabolism (in all disease groups), glutamate metabolism (in OLK and OSCC), taurine and hypotaurine metabolism (in OLP and OLK), and the glucose–alanine cycle. OLK shared more metabolic features with OSCC than with OLP.

**Conclusions:**

Salivary metabolomic profiling reveals distinct metabolic alterations across OPMDs and OSCC, supporting their potential continuum and reflecting disease-related biochemical reprogramming. These findings highlight salivary metabolomics as a promising, non-invasive tool for understanding pathogenesis and for future development of biomarkers.

**Clinical relevance:**

Divergent metabolic pathways in OLP and OLK may relate to their different pathogenesis and malignant transformation risks. Improved understanding of these pathways may support early diagnostics and targeted therapeutic strategies.

**Supplementary Information:**

The online version contains supplementary material available at https://doi.org/10.1007/s00784-026-07192-0.

## Introduction

Oral cancer is a critical global health issue affecting estimated 354,864 new cases per year and majority reported from Asian continent [1]. The World Health Organization (WHO) has anticipated a rise in the incidence of oral cancer in the coming years. In 2007, WHO introduced the term Oral Potential Malignant Lesions (OPMDs), presenting a risk of malignancy, including oral leukoplakia (OLK) and Oral lichen planus (OLP) [2]. It has also been suggested that the term OPMD should be replaced with a new term, “potentially premalignant oral epithelial lesions”, PPOEL [2] Nowadays also erythroplakia, oral submucous fibrosis, actinic cheilitis, oral lupus erythematosus, dyskeratosis congenita, oral lichenoid lesions, oral chronic graft-versus-host disease (OVGHD) as well as reverse smoking related palatal changes are classified as OPMD’s.

OLK is a clinical term (not a histological diagnosis) used to describe asymptomatic white, often plaque-like lesions in the oral mucosa, with potential malignant transformation. OLK is the most prevalent OPMD, and its estimated global prevalence is 1.39% varying between 0.12% and 33.33% presenting higher in males, alcohol consumers and smokers [3]. The speckled leukoplakia, nodular, and verrucous leukoplakia is rare, but it presents an increased risk of malignant potential. A retrospective 20-year analysis of proliferative verrucous leukoplakia (PVL) has shown malignant transformation in approximately 50% of patients, suggesting PVL as a progressive, persistent, irreversible, and aggressive lesion [4].

Oral lichen planus (OLP), a chronic inflammatory disease, manifests in the oral mucosa as different type of white lesions that are immune-mediated [5]. It has been discussed and hypothesized that OLP is associated with leukoplakia and might have partially overlapping phases of the precancerous continuum [6]. The molecular mechanism involved in OLP transformation into malignancy is unknown. However, it is presumed to be exerted by the inflammatory infiltrates present in the subepithelial region [7].

Oral squamous cell carcinoma (OSCC) is the most common malignancy of the oral cavity and the sixth most common malignancy in the world [8]. Alcohol, use of tobacco products, betel leaves, areca nut, and genetic factors are the possible risk factors [9]. OPMD or clinically normal mucosa can give rise to malignancy, and the progression can be accelerated in syndromes such as Fanconi anemia, Li Fraumen syndrome, and xeroderma pigmentosum [2]. Recent concepts in OSCC imply that the oral microbiome is an influential factor that modulates the carcinogenic process. Oral microbiome is diverse and hosts more than 700 prokaryotic species on the gingival sulcus, attached gingiva, tongue, cheek, lip, hard and soft palate, and teeth [10]. It is a complex ecological community. The symbiotic balance of the oral microbiome can be disturbed for several reasons. Such disturbances cause oral dysbiosis and have been associated with leukoplakia, lichen planus, as well as with OSCC [11–13]. For example an abundance of *Firmicutes* and *Actinobacteria* was associated with OSCC and an abundance of *Bacteroidetes* with leukoplakia, meanwhile in patients with lichen planus exhibited an abundance of *Prevotella*,* Fusobacterium*,* Porphyromonas*,* Rothia*,* Oribacterium*,* Leptotrichia* and *Candida* has been observed [14–16].

Saliva assay has a great diagnostic potential in reflecting the metabolite profile (produced by oral microorganisms) non-invasively. NMR spectroscopy of stimulated saliva samples has previously been used to investigate salivary metabolic profiles of patients with OLP [5], as well as OSCC [17]. OLP patients’ salivary metabolic profiles highlighted the elevated levels of acetate, methylamine, and pyruvate, while the concentration of tyrosine was decreased compared to healthy individuals [5]. In patients with head and neck cancer, while undergoing radiotherapy treatment, 17 different salivary metabolites were identified. Most of the metabolites were decreased in patients undergoing treatment, with a few showing elevated pyruvate levels [15]. For the time being, salivary metabolic profiles of OSCC and OLK as well as OSCC and OLP have been studied separately, and have established differences between their profiles [16].

Salivary metabolomic studies in OPMDs and OSCC have mainly been conducted using mass spectrometry. To our knowledge, this study is the first to provide oral metabolic pathways and early biomarkers in OPMDs and OSCC using nuclear magnetic resonance (NMR) spectroscopy. The present study aimed to investigate the salivary metabolites in patients with OLP and OLK and compare the salivary metabolic profiles and pathways in patients with OSCC.

## Materials and methods

The study was approved by the Research Ethics Committee of theNorthern Savo Hospital District (754/31.07.2018 and 22.04.2025), and conducted in accordance with theWorld Medical Association Declaration (Helsinki, Finland, 1964). All participants provided written informed consent form during their clinical visit and before participating in this study.

### Subjects

Sixty participants were enrolled for the study and examined clinically. Diagnoses of OLK, OLP, and OSCC were confirmed by histopathological examination. The cohort included 15 subjects per group: 15 HCs, 15 OLK patients, 15 OLP patients, 15 OSCC patients. Patients with infectious disease, diabetes, other malignant tumors, allergic conditions, and patients who received systemic treatment one month before the experiment were excluded. Some participants across disease groups had hypertension or cardiovascular disease.

The demographic details of the study participants are presented in Table 1.

**Table 1:** Demographic data and saliva flow rate of the subjects

	HCs (*n* = 15)	OLP (*n* = 15)	OLK (*n* = 15)	OSCC (*n* = 15)
Age range(mean ± SD)	36–51 years(44 ± 6.1)	41–53 years(46 ± 5.9)	47–58 years(53 ± 4.2)	40–72 years(57 ± 6.8)
Gender (Male/Female)	6 M/9F	3 M/12F	10 M/5F	10 M/5F
Location	-	BM − 15	BM – 8 Gingiva – 5 Alveolus − 2	Tongue – 8 BM – 4 Retromolar area − 3
Saliva flow rate mL/min (mean ± SD)	0.62 ± 0.31	0.55 ± 0.25	0.54 ± 0.19	0.49 ± 0.15*

*HCs* healthy controls, *OLP* oral lichen planus, *OLK* oral leukoplakia, *OSCC* oral squamous cell carcinoma, *BM* Buccal Mucosa; *p < 0.05 compared to HCs

OLK cases were clinically homogeneous in type, and histologically it showed mild epithelial dysplasia. OLP patients were asymptomatic with a clinical appearance of reticular-type OLP. Histopathologic features supportive of OLP include a hyperkeratotic epithelium with a subepithelial inflammatory infiltrate composed of lymphocytes, apoptotic keratinocytes, and irregular acanthosis. Patients with erosive, bullous, or atrophic OLP or those having medication for oral symptoms have been excluded from the study. OSCCs were clinically verified as Tumor-Node-Metastasis (TNM) stage I (T2N0M0) and histologically graded as well-differentiated on incisional biopsy. Saliva samples were collected before any cancer treatment.

### Saliva collection and preparation

Unstimulated whole saliva was collected between 9:00–11:00 AM to minimize interference of the circadian rhythm. Participants refrained from eating, drinking, and toothbrushing for at least one hour before sampling. Saliva was allowed to drain into a sterile container for 15 min. The flow rate (ml/min) was assessed immediately after the collection. If the salivary flow rate was less than 0,3 ml/min, i.e. hyposalivation, the patient was excluded from the study. The samples were transferred in ice to the laboratory and centrifuged at 14 000 xg for 6 min at 4 °C.The supernatants were stored immediately at -80 °C for NMR analysis.

### NMR sample preparation

Saliva samples were thawed overnight in the refrigerator before NMR sample preparation. The thawed saliva samples were gently mixed and centrifuged (3828 x g, 10 min, + 4 °C). Proteins were removed by pipetting 1 ml of the supernatants to centrifugal filters (Sartorius VIVASPIN 2, 3000 MWCO) and centrifuged (3828 x g, ca. 4 h, + 4 °C) until the filtrate had gone through the filter. The centrifugal filters were washed 13 times with milliQ water before being used to remove glycerol. The NMR samples were prepared by mixing 540 µl of saliva filtrate and 60 µl of potassium phosphate buffer (750 mM potassium dihydrogen phosphate, 0.1% sodium azide, 2.9 mM sodium 3-(trimethylsilyl)propionate-2,2,3,3-d4 in deuterium oxide, pH 7.0) in an Eppendorf tube, and 520 µl of the solution was transferred to a 5 mm NMR tube.

### Measurement of ^1^H NMR spectra

The saliva NMR samples were measured with Bruker AVANCE III HD spectrometer (Bruker BioSpin GmbH, Karlsruhe, Germany) operating at 600.22 MHz and equipped with a cryoprobe (Bruker Prodigy TCI 600 S3 H&F-C/N-D-05 Z). The system contained also an automated SampleJet sample changer (set to + 6 °C temperature). The spectra were measured in automation mode using Icon NMR. Two subsequent samples were automatically transferred to SampleJet heater (set to 22.1 °C) to prewarm the samples. Water-suppressed ^1^H NMR spectra were acquired using noesygppr1d pulse sequence at 22.0 °C. The parameters included spectral width (SW), 21.0360 ppm; time domain (TD), 81,920 points; number of scans (NS), 256; acquisition time (AQ), 3.24 s; relaxation delay (d1), 2.5 s and receiver gain (RG), 32.

The spectral data was automatically processed by Topspin 3.7.0 version including line broadening of 0.3 Hz and zero filling yielding 131,072 data points.

### Quantification of saliva ^1^H NMR spectra

The NMR spectra were manually corrected using TopSpin 3.2 software (Bruker BioSpin GmbH). Before Fourier transformations to spectra, the measured free induction decays were multiplied with an exponential window function with a 1.0 Hz line broadening. The metabolite quantification was made with total-line-shape fitting tool in NMR software (PERCH Solutions Ltd, Kuopio, Finland). The PERCH software allows the accurate quantification of identified metabolites even if the signals are overlapping, or the baseline is not linear due to the heavy protein background envelope or overlapping signals [17]. An internal reference compound (tri-methylsilyl-propanoic acid, TSP) with known concentration, was used as an internal standard. For the method validation, pooled saliva sample from 50 healthy volunteers was used as a quality control sample as described in our previous study [5]. Obtained final metabolite concentrations in saliva are described as µmol/l. All the analyses have been done blinded with no background information from the participants.

### Statistical analysis

Because there were not a priori known target metabolites in OLK, OLP and OSCC groups, we first analysed changed metabolites for the pathway analysis. The Shapiro-Wilk test, the values of kurtosis and skewness were calculated to analyze the data for normality distribution. A paired samples t-test was used to compare values of controls (HCs) to the values of three patient groups, and, if the group data points were not normally distributed, a Mann-Whitney U-test was used to compare salivary metabolite concentrations between each disease groups to HCs. The statistical significance was set at *p* < 0.05. All statistical analyses were conducted using SPSS software, version 27.0 (IBM Corp., Armonk, NY, USA).

To clarify which metabolites, when considering together, give maximum discrimination power between the groups, we used multivariate discrimination function analysis (DFA, Discriminant Function Analysis | SPSS Data Analysis Examples (ucla.edu)). DFA analyses were performed as follows: initially all salivary metabolites having no more than one missing value were entered; and secondly, those metabolites that had no significant correlation with the best single predictor were entered.

To get insight into metabolic mechanisms in different disease groups, the metabolite enrichment analysis was conducted on target metabolites using the Enrichment Analysis-module of MetaboAnalyst 6.0 platform [18]. Pathways were compared across disease groups to identify overrepresented metabolic alterations. The significantly changed metabolites were tested against annotated pathways in the reference metabolome to detect compared over-representations. The statistical analyses of different pathways were based on Fisher’s exact test. False discovery rate (FDR) correction was applied, and a level of *p* < 0.05 was considered statistically significant.

## Results

In all patients, the diagnoses were made on clinical examination and further confirmed histologically. The saliva flow rate was normal for all participants, only for OSCC patients it was slightly decreased. Further, the majority of OSCC patients smoked and OLK patients only three, while there were no smokers in the the OLP and HCs. The demographic data of the study participants and the salivary flow rate are presented in Table 1.

A total of 23 salivary metabolites is detected in the four study groups (Table 1S). Concentrations of two metabolites were below the detection limit: histidine in HCs, and 1,2-propanediol in OLP patients. Of 23 metabolites, five metabolites (acetate, taurine, pyruvate, methylamine and choline) showed elevated concentration whereas proline concentration was decreased in all disease groups when compared to HCs. According to the metabolite analysis, the metabolite profile of OLK patients showed a greater degree of overlap with those of OSCC than with those of OLP. The OSCC group presented elevated levels of 17 metabolites compared to HCs, whereas three metabolites (glycine, proline, and citrate) showed decreased concentration.

In OLP, 11 metabolites exhibited differences compared with HCs (Table 1S). The concentration of butyrate, formate, phenylalnine and tyrosine were lower in OLP patients than in OSCC or HCs. The concentration of 3 metabolites (butyrate, phenylalanine, tyrosine) observed in OLP decreased compared to HCs and OSCC. Similarly, 13 metabolites were detected in the OLK group, of which 10 had elevated concentration when compared to HCs. Only proline concentration was decreased.

Comparison of OSCC and OLK, these groups showed similarly elevated metabolites when compared to HCs, including acetate, alanine, taurine, proline, pyruvate, succinate, methylamine, trimethylamine, 1,2-propanediol, butanol, and fucose. OLP and OLK groups showed differences in taurine, proline, lactate, pyruvate, and methylamine. Acetate, pyruvate and taurine exhibited strong correlations between all disease groups (OLP, OLK & OSCC), and an association with lactate, alanine, trimethylamine, and choline was evident.

**Fig. 1:**
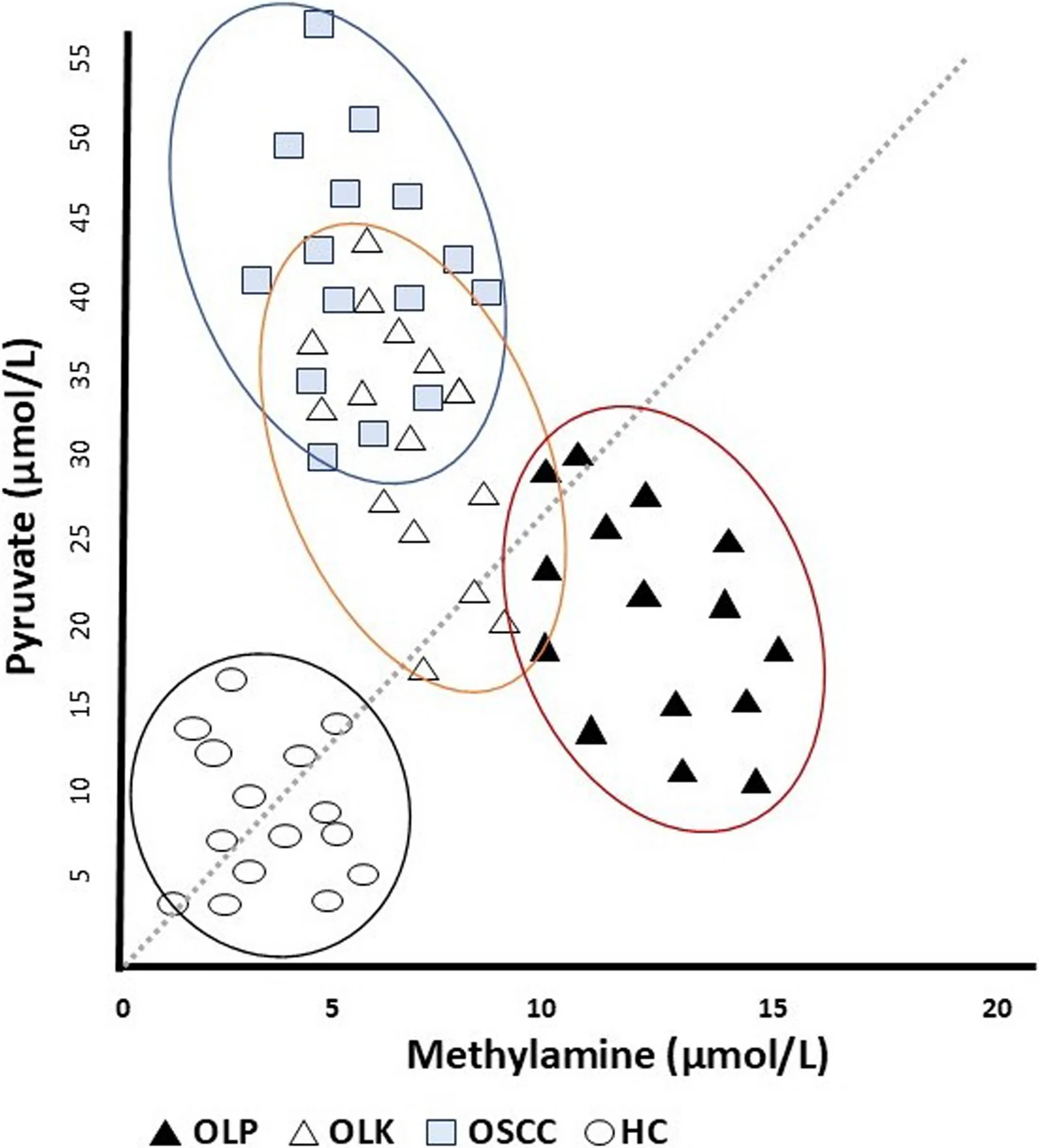
Scatter plot presenting the salivary pyruvate concentrations against salivary methylamine concentrations for HCs and disease groups. (*HC* controls, *OLP* oral lichen planus, *OLK* oral leukoplakia, *OSCC* oral squamous cell carcinoma)

When two metabolites (among the groups of acetate, alanine, taurine, proline, pyruvate, succinate, methylamine, trimethylamine, 1,2-propanediol, butanol, and fucose) were entered into the DFA, a pair of pyruvate and methylamine resulted in the highest discriminant power between HCs and diseases groups: OLP 96.7%; OLK 99.1%; OSCC 99.4% respectively (Fig. 1).

**Fig. 2:**
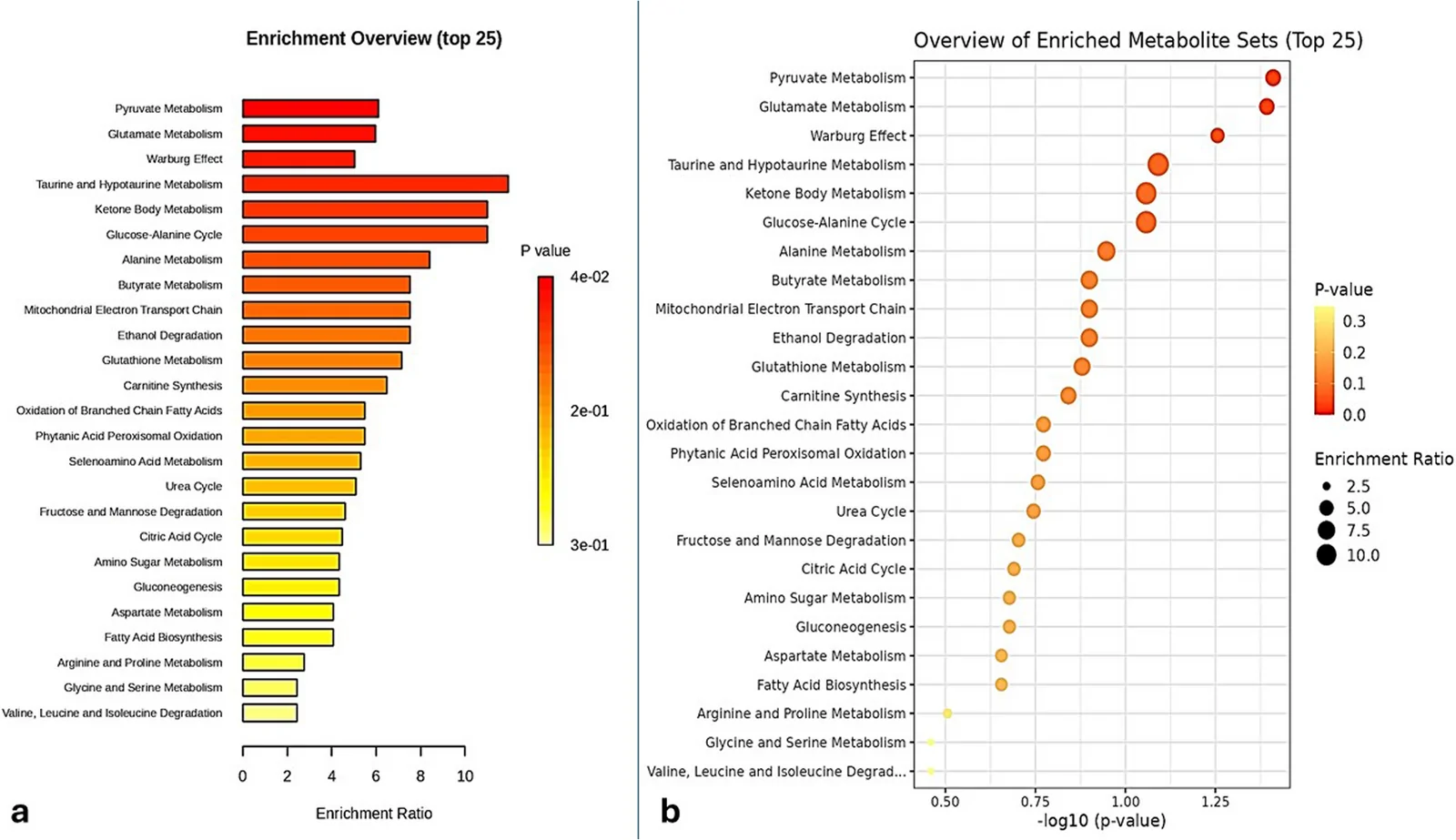
Top 25 predictive metabolic pathways in patients with oral leukoplakia as (**a**) bar chart and (**b**) graphical output (dot plot). The metabolic pathway enrichment analysis revealed two significant pathways: Pyruvate metabolism (*p* = 0.039), and Glutamate metabolism (*p* = 0.041). In addition, three metabolic pathways were almost significantly altered: Warburg effect (*p* = 0.056), Glucose-Alanine Cycle (*p* = 0.088), and Taurine and Hypotaurine Metabolism (*p* = 0.081). Created with MetaboAnalyst 6.0

**Fig. 3:**
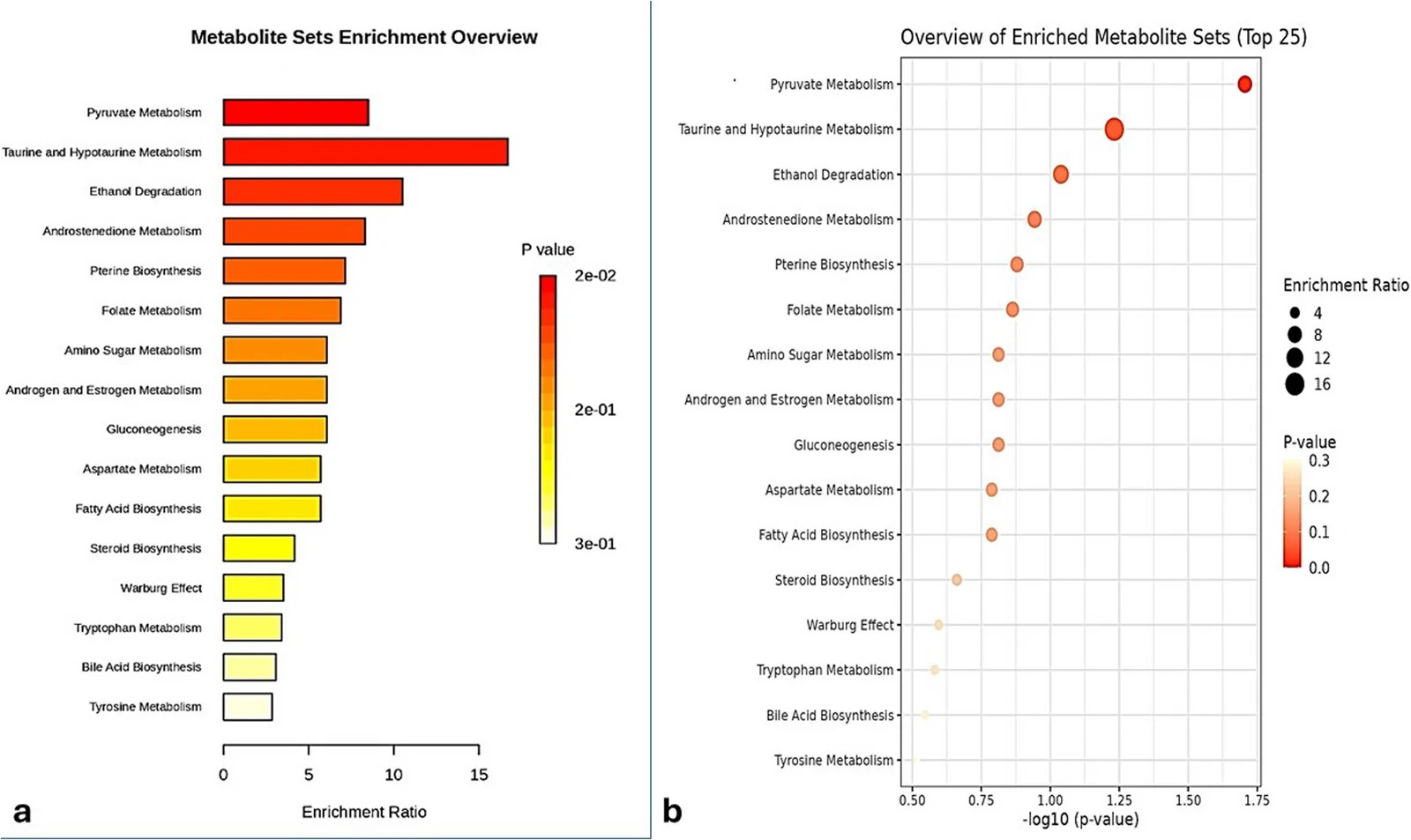
Main predictive salivary pathways in patients with oral lichen planus as (**a**) bar chart and (**b**) graphical output (dot plot). Main changed pathway was Pyruvate metabolism (*p* = 0.020), and Taurine and Hypotaurine Metabolism was almost significant (*p* = 0.059). Created with MetaboAnalyst 6.0

**Fig. 4:**
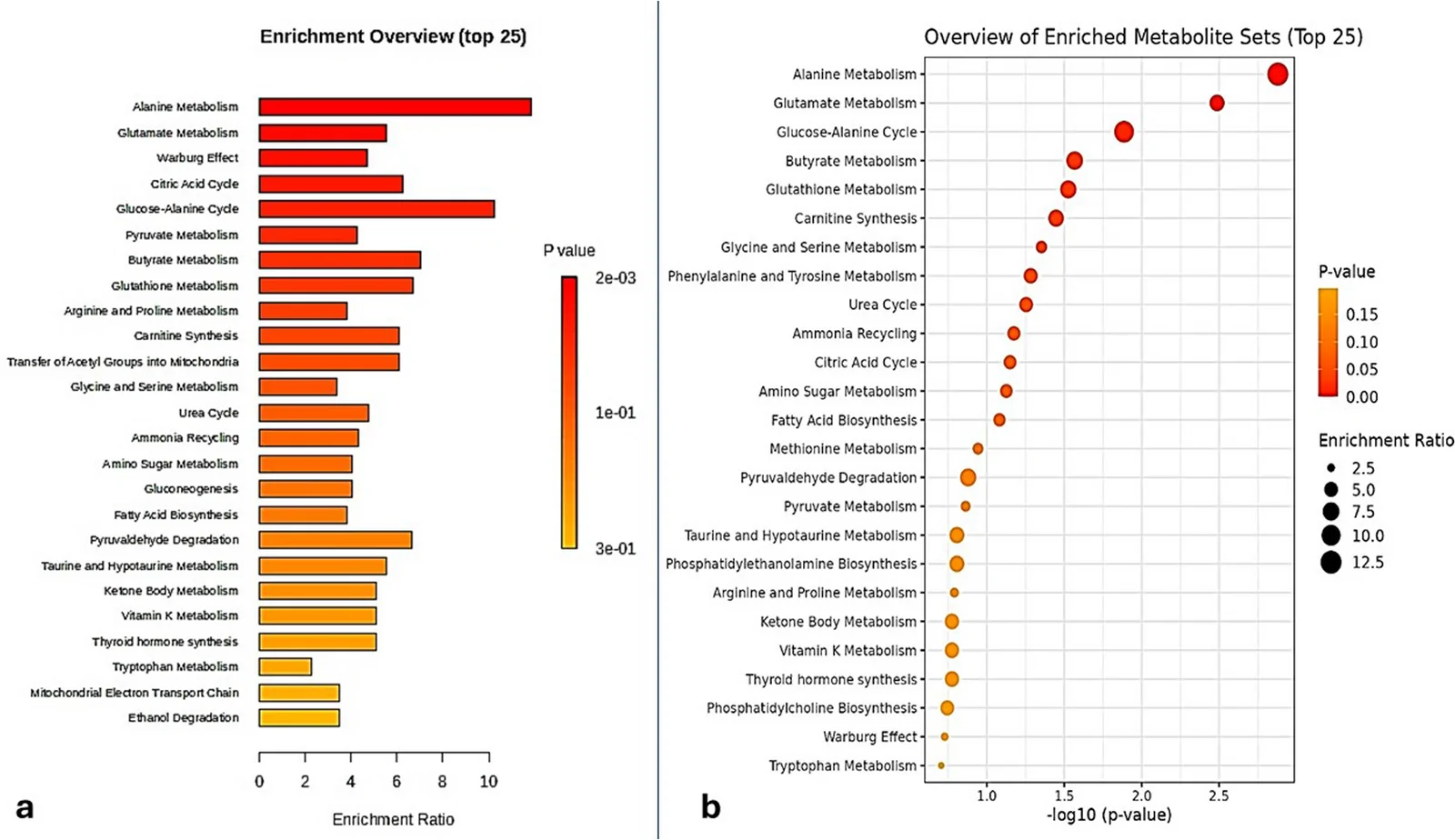
In oral cancer patients, 11 metabolic pathways appeared to be significant as seen in (**a**) bar chart and (**b**) graphical output (dot plot). Changed pathways are also shown in Table 2. Created with MetaboAnalyst 6.0

MetaboAnalyst 6.0, an open-source platform, was used to assess the altered metabolic pathways and their corresponding concentration of metabolites among the disease groups (Figs. 2, 3 and 4). The analysis revealed pyruvate metabolism to be common between OLP, OLK, and OSCC, and aerobic glycolysis (Warburg effect) in OSCC and OLK groups (Table 2).

**Table 2:** List of top 18 pathways identified by pathway enriched analysis, ranked by significance of OSCC pathways. Hits = number of metabolites from experimental data matched to the pathway. Total= total number of compounds in the pathway. p-value (FDR) = significance calculated using Fisher’s exact test and adjusted for multiple tests by false discovery rate. Created by *MetboAnalyst 6.0*

Metabolic pathway	Hits/Total	OSCC *p*-value		OLP *p*-value	OLK *p*-value
Alanine Metabolism	3/17		0.001	-	0.113
Glutamate Metabolism	4/48		0.003	-	0.041
Warburg effects	3/57		0.013	0.254	0.056
Citric Acid Cycle	2/23		0.019	-	0.204
Glucose-Alanine Cycle	2/13		0.023	-	0.088
Pyruvate Metabolism	4/47		0.024	0.020	0.039
Butyrate Metabolism	3/19		0.027	-	0.126
Glutathione Metabolism	2/20		0.030	-	0.132
Arginine and Proline Metabolism	2/52		0.032	-	-
Carnitine synthesis	2/22		0.036	-	0.144
Glycine and Serine Metabolism	3/59		0.044	-	-
Urea Cycle	2/28		0.056	-	0.180
Ammonia Recycling	2/31		0.067	-	-
Amino Sugar Metabolism	2/33		0.075	0.154	0.210
Fatty Acid Biosynthesis	2/35		0.083	0.163	-
Taurine and Hypotaurine Metabolism	1/12		-	0.059	0.081
Ethanol Degradation	1/19		-	0.092	0.126
Gluconeogenesis	4/33		-	0.154	0.210

Pyruvate metabolism presented with high significance and impact in the OLP (*p* = 0.02), OLK (*p* = 0.04), and in OSCC (*p* = 0.02) groups. Warburg effect showed their impact in OSCC (*p* = 0.013) and OLK groups (*p* = 0.056). Such alterations reflect the metabolic dysregulation in the oral cavity of the disease groups. Other pathways that are affected are taurine and hypotaurine metabolism in OLK and OLP. In OSCC group, glutathione metabolism (*p* = 0.030), and glycine and serine metabolism (*p* = 0.044) were significant pathways (Table 2).

## Discussion

This cross-sectional study based on NMR spectroscopy was carried out to assess the salivary metabolic pathways in OSCC and OPMD patients. NMR spectroscopic salivary analysis detected target metabolites and certain pathways having the potential to differentiate OLP, OLK, and OSCC patients from HCs. The univariate analysis showed elevated metabolites, including acetate, taurine, pyruvate, choline, and methylamine concentrations, whereas proline was decreased in all disease groups compared to HCs. A combination of pyruvate together with methylamine provided significant discrimination among disease groups and HCs in the multivariant analysis. Furthermore, the salivary metabolites and metabolic pathways provide information on the changed local environment in the oral cavity.

Pyruvate metabolism pathway was significant in all disease groups. Pyruvate, the end product of glycolysis, has been increased in OLP, OLK, and OSCC salivary samples. Such an increase in salivary pyruvate may indicate increased glycolysis. Generally, pyruvate augments several biosynthetic pathways (protein, nucleotide and lipid) by generating ATP via the citric acid cycle [19]. It has been reported that pyruvate can influence nuclear activity and epigenetic modifications within the cell, and it can participate in the synthesis of fatty acids and amino acids anabolically [20]. Pyruvate role in anaerobic glycolysis, the Pasteur effect, is established as a cellular adaptation to hypoxia, and aerobic glycolysis, The Warburg effect refers to the cancerous cells preference to generate energy through aerobic glycolysis instead of oxidative phosphorylation even with sufficient amount of oxygen being present to support uncontrolled proliferation [21]. Elevated level of salivary pyruvate has been correlated with the later Warburg effect in OSCC [22, 23]. We belive that an increase in the oral microorganism that utilizes the glycolysis pathway for its energy and survival, may lead to increased salivary pyruvate in OLP, OLK, and OSCC. The similarity of this trend to previous findings may highlight the potential influence of microbial communities favoring glycolytic energy production, further supporting a link between oral dysbiosis and epithelial transformation. An increased level of salivary pyruvate in OLP patients has already been shown in our previous publication [5]. Of oral key pathogens e.g. *P. gingivalis* has been associated with increased pyruvate production related also to amyloid-β and Alzheimer’s disease [24].

Short-chain fatty acids (SCFAs), particularly acetate, were markedly increased in all disease groups. Acetate’s microbial origin and its role in biosynthetic pathways underscore its relevance as a metabolic indicator of dysbiosis [25]. Elevated lactate in OLP and OLK likely contributes to increased acetate and propionate through bacterial metabolism. lactate is metabolized to acetate and propionate, which raises the levels of these SCFAs in saliva [26]. In the present study, butyrate, formate, and propionate are other SCFAs detected in human saliva. It is suggested that less than 1% of SCFAs are released as microbial metabolites, products from peptide and amino acid fermentation [27]. A systematic review on SCFAs speculated their role on human oral epithelial cells via receptor-mediated pathways or by inhibition of histone deacetylases, which alters the DNA transcription [28]. SCFAs can activate free fatty acid receptors expressed on immune cells, mainly lymphocytes, neutrophils, and monocytes. Hence, they act as immunomodulators [29]. We speculate that the high concentration of acetate, butyrate, and formate in OLP justifies their immunomodulatory action. However, the acetate rise in OLP, OLK, and OSCC signifies dysbiotic changes in the oral cavity. The high amount of SCFA producing by pathogens has previously correlated e.g. with the severity of periodontitis [5] and SCFA’s are important virulence factors in periodontal pockets for key periodontal pathogens such as *Porphyromonas gingivalis*,* Treponema denticola*, *Aggregatibacter actinomycetemcomitans*,* Prevotella intermedia* and *Fusobacterium nucleatum* [30].

Taurine levels were elevated in all disease groups, consistent with its multifaceted role in cellular homeostasis, antioxidation, and immune function. Taurine synthesis occurs from methionine and cysteine, and is involved in many physiological functions, including osmoregulation, antioxidant, calcium metabolism, and membrane stabilization [31]. The recent evidence suggests paradoxical roles of taurine in cancer, where it supports cancer progression through cellular metabolism and tumor niche signalling. On the other side taurine shapes immune responses across both lymphoid and myeloid compartments [32]. The diagnostic, therapeutic, and risk assessment of taurine in the blood serum are still under investigation, and salivary taurine has not been investigated. Few studies have illustrated taurine’s metabolic plasticity, wherein it promotes tumorigenesis via mTOR activation and apoptosis suppression and/or induces tumor cell death via autophagy and p53 signaling [33]. The presence of high salivary taurine in our study samples questions its antioxidant function and favors its supportive role in tumor progression. Although it highlights the OLP’s and OLK’s potential malignant transformation. The mTOR activation, pro- and anti-apoptotic activity, and p53 role have been discussed in several OLK and OLP studies [34–37]. However, the precise contribution of taurine to premalignant progression remains insufficiently defined and warrants further investigation.

Methylamine, a product of bacterial fermentation and choline metabolism, was also elevated. As a precursor of nitrosamine formation, a known carcinogenic process, methylamine may contribute to a microenvironment supportive of malignant transformation. Methylamine produced from choline metabolism is a harmful substrate for the formation of nitrosamines, which is a known carcinogen [38, 39]. Choline appeared high in our OLP, OLK, and OSCC samples, which hints towards a high level of methylamines. As suggested previously, abnormal choline levels indicate cell proliferation dysregulation [40]. The conversion of amines into toxic metabolites like N-nitroso-dimethylamine using sodium nitrite obtained from food products has also been reported [41]. Nitrosamines are known chemical carcinogens in smoked and smokeless forms of tobacco. Tobacco-specific nitrosamines are a strong etiologic factor for premalignant lesions [42]. Although tobacco has a known impact on OPMDs, it is not evaluated in the present study. The increase in methylamines is justified because of diet and oral dysbiosis.

Proline concentrations were uniformly reduced in the disease groups relative to controls. Proline is an endogenous amino acid that plays a significant role in protein biosynthesis, polyamine synthesis, biochemical and physiological cellular processes such as oxidative stress, immune response, and intercellular signaling [43]. High proline concentration in cancer cells is associated with poor histological differentiation and a high clinical stage of malignancy [44]. Proline is used for signaling and as an alternate source of ATP that is stored in collagen, the main component of the extracellular matrix (ECM) [45]. It has also been suggested that the products formed during the quiescent state of the cells may be deposited as ECM [46]. Hence, the quiescent cells store their metabolic substrates (proline) in the ECM, and this could reflect their lower concentration in the saliva of OLP and OLK. However, their lower concentration in OSCC might protect cancer cells from oxidative stress through proline dehydrogenase, which leads to the uptake and reduced production of reactive oxygen species (ROS). A similar result was mentioned by Wei et al. [47]. The possibility that reduced salivary proline may serve as a prognostic indicator for malignant potential in OPMDs is noteworthy but requires validation in longitudinal studies.

There is not a single salivary biomarker but a bunch of biomarkers, which should be taken as hints of pathological pathways behind. Pathway analysis further highlighted pyruvate metabolism, glutamate metabolism, taurine and hypotaurine metabolism, and the glucose–alanine cycle as key altered pathways. The significant perturbation of glycolytic and gluconeogenic pathways suggests increased glucose utilization and metabolic reprogramming characteristic of dysplasia and malignancy. The aberrant pyruvate metabolism in cancer can downregulate p53 and shift the expression of glycolytic enzymes, such as pyruvate kinase, which is the last enzyme of glycolysis regulating and producing energy (ATP) for cells [48]. Further, the Warburg effect showed affected significantly in OLK and OSCC groups. The Warburg effect refers to the cancerous cells preference to generate energy trough aerobic glycolysis instead of oxidative phosphorylation even with sufficient amount of oxygen being present to support uncontrolled proliferation [20].

The other pathways involved are taurine and hypotaurine metabolism, glutathione metabolism, glycine and serine metabolism, and ethanol degradation. The discriminatory salivary metabolites between OLK and OSCC included alanine, succinate, trimethylamine, 1,2-propanediol, butanol, and fucose. Elevated fucose concentration may indicate more local synthesis by altered cells/cancer cells in OLK and OSCC. A similar finding was observed in our previous publications on OSCC [15, 49]. To differentiate between OLP and OSCC, salivary metabolites butyrate, formate, phenylalanine, tyrosine, and choline were changed. A change in tyrosine levels may imply its participation in PI3K/Akt signalling, NF- kB and p53 transcription factor activation, which forms a basis for malignant transformation. This was established in our previous paper [5].

Although salivary metabolomics offers valuable and non-invasive diagnostic potential, certain limitations must be acknowledged in this study, such as: (1) small sample size; (2) the disease groups are obviously different regarding their typical risk factors, such as smoking. Furthermore, in the OLP group, there are only patients who had asymptomatic reticular-type OLP and do not need treatment. In further studies, it is interesting to find out the changes in the metabolic profile between different types of lichen, because OLP manifests clinically in variable forms. However, participants in this study were selected based on clinical and histological criteria that were consistent for all. While smoking and alcohol are known risk factors for oral cancer, a biomarker such as the salivary profile or oral microbiome could help identify a risk indicator or a potential risk factor. Additionally, such biomarkers could help identify patients with OPMDs that are likely to undergo malignant transformation.

Validated salivary metabolomic-based risk scores for OSCC will be applied to identify high-risk individuals. The salivary biomarkers are attractive in oral mucosal diseases, since these lesions communicate with saliva and might lead to the development of specific salivary biomarkers. The current evidence about the diagnostic potential of reported salivary biomarkers in various pathologies is still weak and needs to be strengthen in further validation studies with larger number of samples. The findings of this study will inform future investigations and supports a precision health framework for early targeted intervention before disease manifests.

Differences in the DFA analysis and metabolic pathways between OLP and OLK may reflect their different pathogenesis and malignant transformation risks. Understanding the role of changed salivary metabolic pathways in OPMDs and their role in malignant transformation could help raise awareness for early and planned management, and even the development of specific treatments. Effective metabolomic-based salivary pathways are worthy of further investigation and further research can be used to move to clinically actionable solutions in OPMDs monitoring. This preliminary study provides encouraging results, which could be the basis for further controlled longitudinal trials with more patient samples to ensure the salivary metabolomic true diagnostic accuracy and feasibility or even study as a cancer risk predictor in OPMDs.

## Supplementary Information

Below is the link to the electronic supplementary material.

Supplementary Material 1 (DOCX 51.9 KB)

## Data Availability

No datasets were generated or analysed during the current study.
