# Supplementary material for: Salivary metabolic profile in patients with oral squamous cell carcinoma and potentially malignant disorders
Source: Clin Oral Investig. 2026 Oct 2;30(10):495. doi: 10.1007/s00784-026-07192-0 (PMC13633301; doi:10.1007/s00784-026-07192-0)
Supplement: Supplementary file 1 [file 784_2026_7192_MOESM1_ESM.docx]

| **Metabolite** | **HCs**  **(N=15)** | **OLK patients**  **(N=15)** | **HC/OLK**  **p-value** | **OLP patients**  **(N=15)** | **HC/OLP**  **p-value** | **OSCC patients**  **(N=15)** | **HC/OSCC**  **p-value** | |
| --- | --- | --- | --- | --- | --- | --- | --- | --- |
| **SCFAs** |  |  |  |  |  |  |  | |
| Acetate | **1783.0 (621.3- 2201.3)^b^** | 2133.7 ± 871.2 | **0.008** | 2021.7 (700.1-2399.8) | **0.028** | 2.978,6 (1783.0 - 2715.2) | **0.001** |  |
| Butyrate | **46.4 ± 12.9^a^** | 43.4 ± 22.9 | 0.731 | 33.9 ± 20.9 | **0.015** | 99.3 ± 101.0 | **0.002** |  |
| Formate | **98.9 ± 63.3 ^a^** | 110.0 ± 83.2 | 0.579 | 154.0 ± 121.0 | **0.036** | 229.7 ± 51.7 | **0.004** |  |
| Propionate | **259.2 ± 167.2 ^a^** | 297.7 ± 199.3 | 0.532 | 248.3 ± 195.7 | 0.971 | 777.3 ± 481.4 | **0.000** |  |
| **Amino acids** |  |  |  |  |  |  |  |  |
| Alanine | **12.1 ± 8.8 ^a^** | 56.6 ± 23.0 | **0.004** | 13.8 ± 13.2 | 0.921 | 90.3 ± 31.1 | **0.000** |  |
| Glycine | **522.5 ± 35.4 ^a^** | 520.8 ± 33.9 | 0,988 | 510.9 ± 25.5 | 0.842 | 460.8 ± 84.9 | **0.048** |  |
| Phenylalanine | **68.5 ± 3.6 ^a^** | 57.8 ± 12.8 | 0.371 | 19.3 ± 5.4 | 0.009 | 99.4 ± 23.1 | **0.032** |  |
| Taurine | **48.3 ± 29.0 ^a^** | 102.5 ± 55.6 | **0.013** | 121.5 ± 84.5 | **0.000** | 132.4 ± 87.1 | **0.000** |  |
| Proline | **579.1 ± 154.1 ^a^** | 158.3 ± 129.9 | **0.015** | 143.8 ± 127.5 | **0.005** | 182.8 ± 151.2 | **0.018** |  |
| Histidine | **#** | 20.7 ± 19.1 | - | 25.31 ± 10.9 | - | 24.3 ± 12.8 | # |  |
| Tyrosine | **87.6 ± 21.4 ^a^** | 99.8 ± 32.5 | 0.549 | 44.9 ± 21.3* | **0.047** | 125.8 ± 27.2* | **0.028** |  |
| **Organic acids** |  |  |  |  |  |  |  |  |
| Citrate | **18.3 (8.5-27.2)^b^** | 19.3 (8.3-34.4) | 0.923 | 21.1 (6.8-30.8) | 0.721 | 12.0 ± 8.9 | **0.037** |  |
| Lactate | **201.3 (134.5-327.0) ^b^** | 288.5 (120.1-507.7) | **0.047** | 275.1 (141.3-287.2) | **0.050** | 207.5 ± 134.7 | 0.788 |  |
| Pyruvate | **11.6 ± 7.8 ^a^** | 30.2 ± 15.2 | **0.004** | 21.6 ± 12.9 | **0.041** | 38.1 ± 29,0 | **0.001** |  |
| Succinate | **38.2 ± 16.2 ^a^** | 55.3 ± 21.1 | 0.048 | 29.8 ± 20.6 | 0.472 | 64.9 ± 19.8 | **0,019** |  |
| **Amines** |  |  |  |  |  |  |  |  |
| Methylamine | **2.5 ± 0.8 ^a^** | 5.9 ± 3.3 | **0.019** | 14.1 ± 1.8 | **0.000** | 6.2 ± 1.9* | **0.005** |  |
| Trimethylamine | **1.6 ± 1.2 ^a^** | 15.8 ± 2.7 | **0.002** | 1.4 ± 1.4 | 0.859 | 20.1 ± 1.8 | **0.000** |  |
| **Other** |  |  |  |  |  |  |  |  |
| Choline | **6.2 ± 2.1 ^a^** | 9.8 ± 6.3 | 0.118 | 10.1 ± 4.3 | **0.037** | 20.9 ± 10.4 | **0.009** |  |
| Isopropanol | **2.5 ± 1.3 ^a^** | 3.1 ± 0.8 | 0.878 | 2.5 ± 0.9 | 0.998 | 3.7 ± 2.2 | 0.621 |  |
| 1.2-propanediol | **29.3 (15.6-53.8) ^b^** | 61.3 (22.9-190.0) | **0.030** | # | - | 58.3 (19.8-91.1) | **0.049** |  |
| Butanol | **19.6 ± 11.3 ^a^** | 37.8 ± 29.5 | **0.024** | 22.1 ± 8.1 | 0.722 | 71.28 ± 52.7 | **0.009** |  |
| Methanol | **27.7 ± 9.2 ^a^** | 34.2 ± 19.9 | 0.194 | 29.1 ± 19.5 | 0.802 | 99.2 ± 5.7 | **0.001** |  |
| Fucose | **86.7 ± 51.2 ^a^** | 663.2±131.0 | **0.000** | 54.6 ± 29.8 | 0.235 | 783.3 ± 120.9 | **0.000** |  |

**Table 2.** Salivary metabolite concentrations in pateints with oral leukoplakia (OLK), oral lichen planus (OLP), oral squamous cell carcinoma (OSCC) and oral squamous cell carcinoma are compared to healthy controls (HCs).

A paired samples t-test [mean ± std. deviation (a)] was used to compare HCs to OPMDs and OSCC, if the differences between HCs and disease groups were not normally distributed Mann Whitney U-test [median (minimum‒maximum) (b)] was used.
